# TelomereHunter – in silico estimation of telomere content and composition from cancer genomes

**DOI:** 10.1186/s12859-019-2851-0

**Published:** 2019-05-28

**Authors:** Lars Feuerbach, Lina Sieverling, Katharina I. Deeg, Philip Ginsbach, Barbara Hutter, Ivo Buchhalter, Paul A. Northcott, Sadaf S. Mughal, Priya Chudasama, Hanno Glimm, Claudia Scholl, Peter Lichter, Stefan Fröhling, Stefan M. Pfister, David T. W. Jones, Karsten Rippe, Benedikt Brors

**Affiliations:** 1 Division of Applied Bioinformatics, German Cancer Research Center (DKFZ), 69120 Heidelberg, Germany; 2 Faculty of Biosciences, Heidelberg University, 69120 Heidelberg, Germany; 3 Research Group Genome Organization & Function/Division of Chromatin Networks, German Cancer Research Center (DKFZ) and BioQuant Center, 69120 Heidelberg, Germany; 4 Division of Theoretical Bioinformatics, German Cancer Research Center (DKFZ), 69120 Heidelberg, Germany; 5 Division of Pediatric Neurooncology, German Cancer Research Center (DKFZ), 69120 Heidelberg, Germany; 6 Division of Translational Medical Oncology, National Center for Tumor Diseases (NCT) Heidelberg and German Cancer Research Center (DKFZ), 69120 Heidelberg, Germany; 7 Translational Functional Cancer Genomics, National Center for Tumor Diseases (NCT) and German Cancer Research Center (DKFZ), Heidelberg, Germany; 8 Department of Translational Medical Oncology, National Center for Tumor Diseases (NCT) Dresden, University Hospital Carl Gustav Carus, Dresden and DKFZ, Heidelberg, Germany; 9 German Cancer Consortium (DKTK), 69120 Heidelberg, Germany; 10 Division of Applied Functional Genomics, DKFZ, 69120 Heidelberg, Germany; 11 Division of Molecular Genetics, German Cancer Research Center (DKFZ), 69120 Heidelberg, Germany; 12 Hopp Children’s Cancer Center at the NCT Heidelberg (KiTZ), 69120 Heidelberg, Germany; 13 Department of Pediatric Oncology, Hematology and Immunology, University Hospital Heidelberg, 69120 Heidelberg, Germany; 14 Pediatric Glioma Research Group, German Cancer Research Center (DKFZ), 69120 Heidelberg, Germany

## Abstract

**Background:**

Establishment of telomere maintenance mechanisms is a universal step in tumor development to achieve replicative immortality. These processes leave molecular footprints in cancer genomes in the form of altered telomere content and aberrations in telomere composition. To retrieve these telomere characteristics from high-throughput sequencing data the available computational approaches need to be extended and optimized to fully exploit the information provided by large scale cancer genome data sets.

**Results:**

We here present TelomereHunter, a software for the detailed characterization of telomere maintenance mechanism footprints in the genome. The tool is implemented for the analysis of large cancer genome cohorts and provides a variety of diagnostic diagrams as well as machine-readable output for subsequent analysis. A novel key feature is the extraction of singleton telomere variant repeats, which improves the identification and subclassification of the alternative lengthening of telomeres phenotype. We find that whole genome sequencing-derived telomere content estimates strongly correlate with telomere qPCR measurements (r = 0.94). For the first time, we determine the correlation of in silico telomere content quantification from whole genome sequencing and whole genome bisulfite sequencing data derived from the same tumor sample (r = 0.78). An analogous comparison of whole exome sequencing data and whole genome sequencing data measured slightly lower correlation (r = 0.79). However, this is considerably improved by normalization with matched controls (r = 0.91).

**Conclusions:**

TelomereHunter provides new functionality for the analysis of the footprints of telomere maintenance mechanisms in cancer genomes. Besides whole genome sequencing, whole exome sequencing and whole genome bisulfite sequencing are suited for in silico telomere content quantification, especially if matched control samples are available. The software runs under a GPL license and is available at https://www.dkfz.de/en/applied-bioinformatics/telomerehunter/telomerehunter.html.

**Electronic supplementary material:**

The online version of this article (10.1186/s12859-019-2851-0) contains supplementary material, which is available to authorized users.

## Background

Telomeres are nucleoprotein complexes at the ends of eukaryotic chromosomes. In humans, telomeric DNA consists mainly of non-coding t-type (TTAGGG) repeats. However, c- (TCAGGG), g- (TGAGGG) and j-type (TTGGGG) telomeric variant repeats (TVRs) as well as other variations of the hexameric sequence exist [1–3]. Telomeres shorten with each cell division [4] and once a critical telomere length is reached, a DNA damage response is triggered, resulting in cellular senescence or apoptosis [5, 6].

To circumvent the limited number of possible cell divisions, tumors employ activation of telomerase [7] or alternative lengthening of telomeres (ALT) [8] as telomere maintenance mechanisms (TMMs). Telomerase is an enzyme that adds t-type repeats to the chromosome ends [9]. In contrast, ALT is based on recombination of telomeric regions and results in several characteristics, including telomeres of heterogeneous length [8] and sequence composition [3, 10].

These TMMs are crucial for tumorigenesis, making them valuable drug targets for cancer therapy [11]. However, to precisely identify and interfere with these mechanisms in various tumor types, more insight into the different telomere structures is needed. In the last decades, several experimental methods have been established to assess telomere length and ALT status, e.g. telomere qPCR, terminal restriction fragment (TRF) analysis and C-circle assay [12, 13].

With the advance of massively parallel sequencing, an alternative method for measuring telomere content has emerged. Several studies showed that the number of short reads containing telomeric repeats can be used to estimate telomere content in whole genome sequencing (WGS) data, yielding results comparable to those of established experimental methods [10, 14–18]. This type of analysis yields valuable insight into telomeric features in cancer data as described in several recently published cancer studies [19–21]. Here, we present TelomereHunter, a new computational tool for determining telomere content that is specifically designed for matched tumor and control pairs. In contrast to existing tools, TelomereHunter takes alignment information into account and reports the abundance of variant repeats in telomeric sequences. We introduce the main features of TelomereHunter, discuss the interpretation of exemplary results for ALT-positive and ALT-negative tumor samples, characterize the tool in comparison to biological assays for telomere content estimation and assess the impact of different sequencing protocols on the telomere content quantification.

## Results

### Software features

**Fig. 1:**
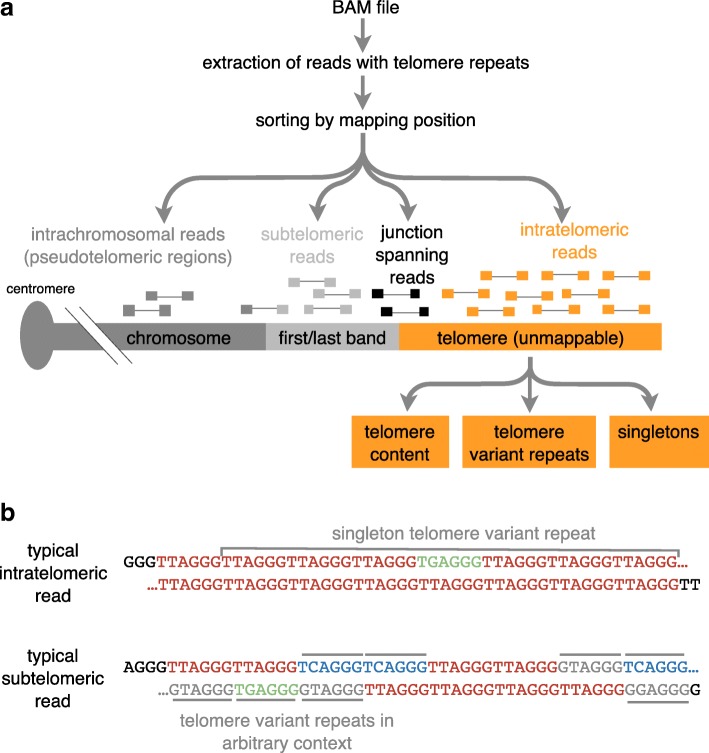
TelomereHunter workflow. **a** TelomereHunter extracts reads containing telomere repeats from an input BAM file. The reads are sorted by mapping position into intrachromosomal, subtelomeric, junction spanning and intratelomeric reads. From the intratelomeric reads, telomere content, telomere variant repeats and singletons are obtained. **b** Examples of a typical intratelomeric read containing a TGAGGG singleton and a typical subtelomeric read containing multiple telomere variant repeats in arbitrary context

In the first analysis step, TelomereHunter extracts reads with a high telomeric repeat content from next-generation sequencing data in BAM format. The pre-configured selection criteria use a threshold of at least six t-type, c-type, g-type or j-type hexameric repeats or their reverse complements to classify a 100 bp long read as telomeric. This threshold is automatically adjusted for other read lengths. The selection threshold as well as the search patterns are adaptable. Furthermore, non-consecutive (default) or consecutive appearance of these search patterns can be configured. In the second step, alignment information from the BAM file is applied to subclassify selected reads into the four categories: intratelomeric, junction spanning, subtelomeric and intrachromosomal (Fig. 1a).

The intratelomeric reads are further analyzed to quantify their TVR content and the presence of singletons, which are TVRs embedded in canonical t-type repeats. This analysis of the singleton TVR count is instrumental for distinguishing telomeric from unmappable subtelomeric reads (Fig. 1b).

To calculate the normalized GC-corrected telomere content for a BAM file as previously described by Ding et al. [14], the number of intratelomeric reads is normalized by the number of reads of comparable GC content (48–52%) and multiplied by 10^6^, a unit that we abbreviated by TRPM (telomeric reads per GC content-matched million reads). If a matched control sample is available, the telomere content tumor/control log2 ratio (*log*_*2*_ *T/C*) is computed.

**Fig. 2:**
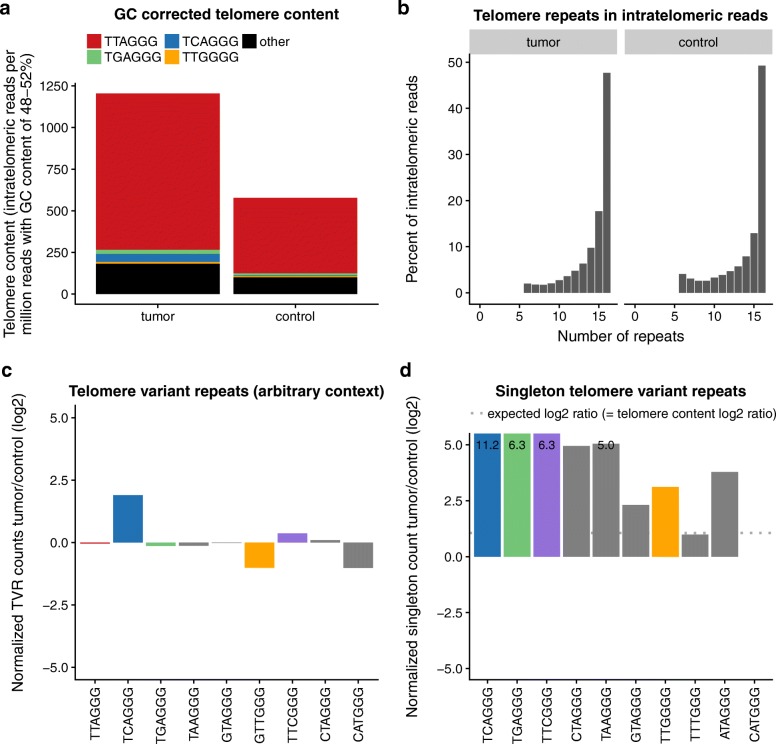
Exemplary ALT-positive glioblastoma patient. **a** GC corrected telomere content: Telomere content of tumor and control sample in reads per million GC-matched reads. Contributions of TVRs are indicated by color code. **b** Telomere repeats in intratelomeric reads: Histogram of repeat units per read for all extracted intratelomeric reads. **c** Telomere variant repeats (arbitrary context): Overview on *log*_*2*_ *T/C* of TVRs in arbitrary context. **d** Singleton telomere variant repeats: Overview on *log*_*2*_ *T/C* of TVRs in singleton context

**Fig. 3:**
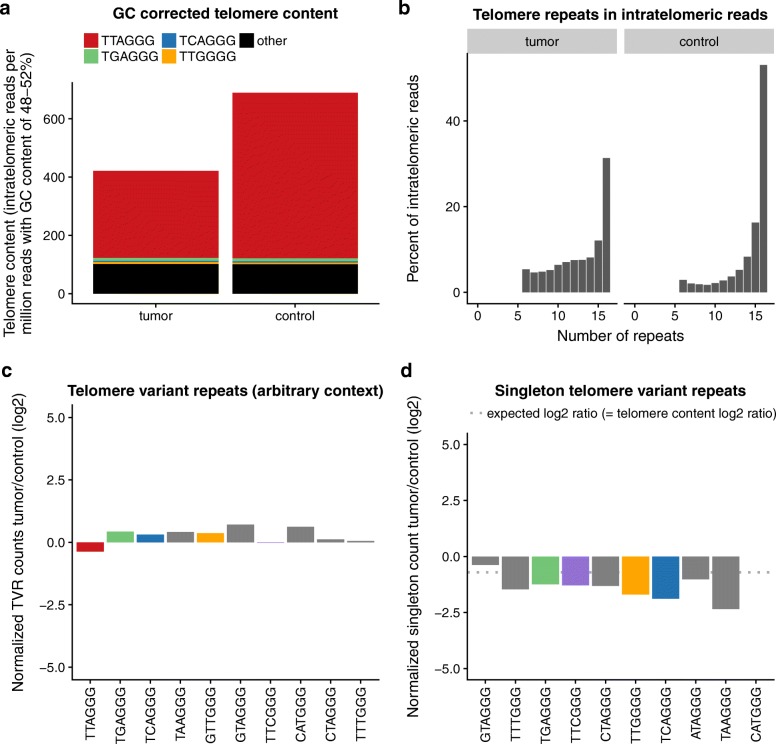
Exemplary ALT-negative medulloblastoma patient. **a** GC corrected telomere context: Telomere content of tumor and control sample in reads per million GC-matched reads (RGCM). Contribution of TVR is indicated by color code. **b** Telomere repeats in intratelomeric reads: Histogram of repeat units per read for all extracted intratelomeric reads. **c** Telomere variant repeats (arbitrary context): Overview on *log*_*2*_ *T/C* of TVRs in arbitrary context. **d** Singleton telomere variant repeats: Overview on *log*_*2*_ *T/C* of TVRs in singleton context

Next, the tool performs a comprehensive analysis of the determined TVR count of tumor and matched control samples specifically for intratelomeric reads, illustrated here by an ALT-positive (Fig. 2) and an ALT-negative (Fig. 3) sample. This comprises the contribution of t-type and TVRs to the GC-corrected telomere content (Figs. 2a and 3a), the distribution of telomeric hexamer counts per intratelomeric read (Figs. 2b and 3b), the *log*_*2*_ *T/C* of TVRs in arbitrary context (Figs. 2c and 3c) and of singleton TVRs (Figs. 2d and 3d).

It is noteworthy that a considerable number of reads containing TVRs are frequently classified as intrachromosomal, usually originating from pseudotelomeric regions. To support the analysis of these regions, the distribution of subtelomeric, junction spanning and intrachromosomal reads across individual chromosomes are summarized in an additional diagram (Additional file 1: Figure S1). By using aligned reads (BAM files) instead of unaligned reads (FASTQ files) as input to TelomereHunter, this potential noise is removed from the analysis of intratelomeric reads. Thereby, the detection of ALT-characteristic aberrations in the TVR distribution is improved.

A complete overview of all parameters as well as the generated visualizations and data files is given in Additional file 1: Tables S1 and S2, respectively. The run time of TelomereHunter depends on the size of the BAM file (Additional file 1: Table S3). The software and documentation is available at https://www.dkfz.de/en/applied-bioinformatics/telomerehunter/telomerehunter.html.

### Application of TelomereHunter

As an exemplary application of TelomereHunter we compared an ALT-positive and an ALT-negative case from the ICGC PedBrain project. Based on C-circle and TRF assays, patient GBM56 [22] was classified as an ALT-positive glioblastoma case, in which alterations of telomere content and composition were particularly pronounced. The GC-corrected telomere content was doubled in the tumor samples compared to the matched control. Our analysis reveals that this extension of telomere repeats is due to an increase of the canonical t-type repeats, and a gain of particular TVRs (Fig. 2a). No pronounced shift in the frequency of hexameric repeats per telomeric read was detected (Fig. 2b). While only the c-type repeat *log*_*2*_ *T/C* was increased among the TVRs in arbitrary context (Fig. 2c), many singleton TVRs were strongly enriched in the tumor sample (Fig. 2d). Recombination events that span the t-type rich telomeres as well as part of the proximal subtelomeric region could rationalize these observations, as for instance shown in Varley et al. [3].

In contrast, the tumor sample of medulloblastoma patient MB79 [23] was ALT-negative according to C-circle assay and TRF analysis. A characteristic moderate decrease of telomere content in the tumor sample was found in our analysis, which was accompanied by a stable TVR count (Fig. 3a). This reflects a shortening of the telomeres, while the TVR-containing subtelomeric regions remained unaltered. Likewise, the relative number of hexameric telomere repeats per read is reduced, which is characteristic for subtelomeric genome regions that are relatively enriched through the loss of distal telomeric sequence (Fig. 3b). In consequence, a moderate gain of TVRs in arbitrary context was observed, while the t-type *log*_*2*_ *T/C* was negative (Fig. 3c) and the number of singleton TVRs was reduced (Fig. 3d). A more comprehensive characterization of telomeric features in cancer data can be found in recently published pan-cancer studies [19–21].

### Characterization of software

We characterized the TelomereHunter-based telomere content quantification by comparing it to established experimental methods for telomere content measurement. Nine pediatric brain tumor samples (six medulloblastoma and three glioblastoma samples) were sequenced by whole genome sequencing (WGS). Subsequently, the telomere content was determined computationally by TelomereHunter and also measured by telomere qPCR and TRF analysis. We included samples with different ALT status into the analysis (as determined by TRF and C-circle assay, Additional file 1: Figure S2) to assess if the TelomereHunter approach determines the telomere content of both ALT-positive and ALT-negative samples with high concordance to biological assays.

The experimentally determined telomere content estimation matched well with the TelomereHunter results (Additional file 1: Figure S3a) and was highly correlated for the individual tumor and control samples (r = 0.90 for qPCR and r = 0.65 for TRF, Pearson correlation). The correlation was further improved by GC correction of the computationally determined telomere content (r = 0.94 and 0.72, Pearson correlation) (Additional file 1: Figure S3b). The Pearson correlation of qPCR to TRF measurements was r = 0.65 (individual tumor and control samples) and r = 0.83 (*log*_*2*_ *T/C*). It has been observed that several tools for telomere content estimation from cancer genome data show a comparable performance [18]. We confirmed this by benchmarking four software tools in addition to TelomereHunter (Additional file 1: Figure S3c-d). For all tools, the *log*_*2*_ *T/C* correlated better with the experimental measurements than the direct comparison of unmatched samples. TRF and qPCR correlate better with most of the software predictions than with each other.

The unique alignment-based classification of extracted reads performed by TelomereHunter filters intratelomeric from chromosomal telomere reads. While the impact of this filtering step is relatively minor in samples with high telomere content, more than 25% of telomeric reads are aligned to unique genome regions and thus removed from telomere content estimation in samples with low telomere content (Additional file 1: Figure S4). Notably, the majority of intratelomeric reads are aligned with a mapping quality of 0 using the alignment algorithm bwa-mem (Additional file 1: Figure S5).

### NGS protocol and computational preprocessing comparison

**Fig. 4:**
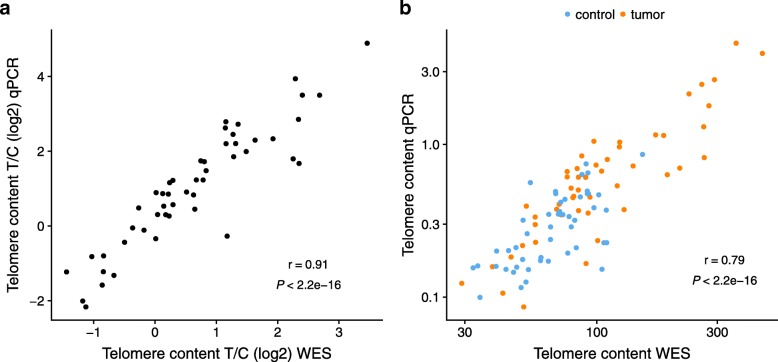
Telomere content estimation from WES data. **a** Correlation of telomere content *log*_*2*_ *T/C* determined by telomere qPCR and TelomereHunter for WES samples aligned with bwa-mem from 49 leiomyosarcoma patients. **b** Correlation of qPCR and TelomereHunter results for individual tumor and control samples of the same patients (shown on a logarithmic scale). The Spearman correlation coefficients are indicated

In cancer research, patient DNA is analyzed by various sequencing protocols, such as exome sequencing or special chemical modifications, for instance to assess cytosine methylation through bisulfite treatment. We here quantified the impact of two such sequencing protocols on the results of TelomereHunter. First, we selected 49 leiomyosarcoma tumor/control sample pairs for which WES data and telomere content estimations by qPCR were available [24]. The comparison showed a highly significant correlation (r = 0.91, *p* < 2.2·10^− 16^, Spearman correlation of bwa-mem aligned samples) between the *log*_*2*_ *T/C*, but a reduced correlation (r = 0.79, p < 2.2·10^− 16^, Spearman correlation) for the individual tumor and control samples (Fig. 4). While the analyzed tumor and matched control samples were sequenced simultaneously, overall sequencing of the test cohort was conducted at different time points and thus under slightly varying conditions. Our observations imply that these batch effects can result in an increased technical variability when using WES protocols for direct quantification, but are partially canceled out by taking the *log*_*2*_ *T/C*. We then used the leiomyosarcoma cohort to test the influence of alignment algorithms, the alignment filter and the focus on t-type repeats during read extraction on the telomere content estimation (Additional file 1: Figure S6). In summary, telomere content *log*_*2*_ *T/C* estimated from the different alignment algorithms bwa-mem and bwa-aln [25, 26] correlate well in a matched tumor control setting (r = 0.99). In an unmatched or control-free setting, the impact of the alignment algorithm is more pronounced (r = 0.95). More specifically, preprocessing with the bwa-mem algorithm results in telomere content estimates that correlate better with q-PCR-based telomere content measurements (*log*_*2*_ *T/C*: r = 0.91; direct: r = 0.79) than is the case with the bwa-aln algorithm (*log*_*2*_ *T/C*: r = 0.90*;* direct: r = 0.72). Next, including uniquely aligned telomeric repeats into the estimation decreased the correlation from 0.91 to 0.90 for *log*_*2*_ *T/C* and from 0.79 to 0.73 in the direct setup. In contrast, a focus on t-type repeats actually improved the respective correlation to 0.92 and 0.81, and thus is beneficial in a setting where telomere content analysis is favored over TVR profiling. Furthermore, the use of different versions of the reference genome sequence had a minor impact on the estimation (Additional file 1: Figure S7).

The influence of the repeat threshold parameter on the telomere content estimation was tested using the leiomyosarcoma WES data and the nine WGS brain cancer samples (Additional file 1: Figure S8). The analysis showed that for WGS and WES data the default parameter of 6 repeats per 100 bp read length and higher parameter choices produce a robustly good correlation with qPCR-based measurements. In contrast, lower threshold choices reduce the correlation, thus affecting the WES data more strongly than the WGS data.

**Fig. 5:**
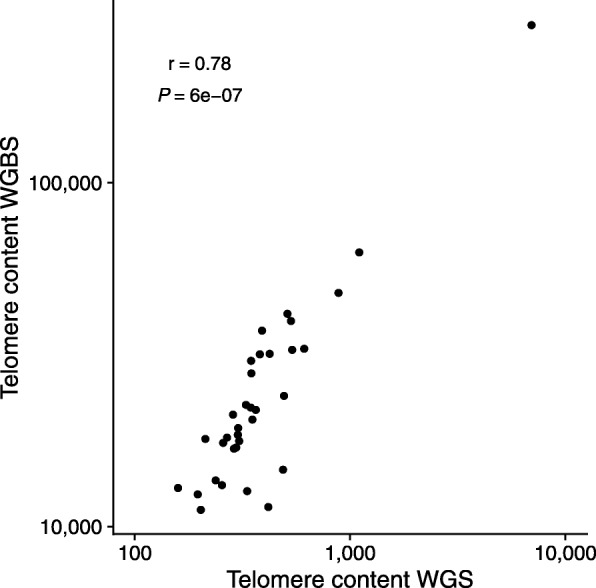
Telomere content estimation from WGBS data. Correlation of telomere content estimation from WGBS and WGS data of 34 medulloblastoma samples using TelomereHunter. The Spearman correlation coefficient is indicated

Next, we applied TelomereHunter to 34 medulloblastoma samples, which were sequenced by WGS as well as using a WGBS protocol [27]. The bisulfite treatment converts unmethylated cytosine to uracil and then to thymine during DNA library preparation. Because the cytosines of the dominant t-type repeats are unmethylated, the complexity of the antisense strand is reduced from CCCTAA to TTTTAA, which is not specific enough to be considered during telomere content quantification. The sense strand is unaffected due to the absence of cytosine. When applied to sequencing data produced by WGBS, TelomereHunter therefore depends exclusively on information from the telomeric sense strand. The telomere content analysis showed a correlation of WGS (aligned with bwa-mem) and WGBS data (aligned with bwa-aln) that surpasses the WGS/WES correlation for individual samples (r = 0.78, *p* = 6.0·10^− 7^, Spearman correlation) (Fig. 5). For the WGBS cohort, the absolute number of extracted intratelomeric reads was 2.9-fold increased compared to the WGS data (3.1-fold increase if corrected for sequencing depth). This result is counter-intuitive, given the loss of information from the telomeric antisense strand. We speculate that the absence of cytosines in the telomeric sense strand protects telomeric sequences from DNA damage during bisulfite treatments, and thus leads to a relative enrichment of the telomeric fraction during DNA library preparation. At the same time, the relative amount of reads with a GC content around 50% is twentyfold lower in WGBS data due to the cytosine conversion (0.6% of all reads in WGBS as compared to 12% in WGS). Together this leads to higher telomere content values in WGBS data (mean = 31,782 TRPM) compared to WGS data (mean = 580 TRPM). Despite the differences in numeric range, these results show that telomere content estimations from WGBS genomes are at least as reliable as quantification from WES data in a control-free study design.

## Discussion

TelomereHunter’s main purpose is the efficient analysis of telomeric sequences in matched case and control genomes. It produces an array of diagnostic diagrams, which allow a detailed characterization of the sample’s telomere content and composition. It furthermore provides results in machine readable format and enables an easy aggregation of data from multiple samples into cohort studies. Finally, the extracted and subclassified telomeric reads are provided as individual BAM files for customized follow-up analysis.

A currently unique feature of the tool is the usage of aligned reads for deconvolution of the different genomic sources of telomere-repeat-containing reads. Furthermore, the distinction of TVRs in singleton context from mere TVR quantification increases the information content of the results. This addition is of special relevance for the study of the ALT phenotype as demonstrated in a recent pan-cancer study [21].

Computational telomere content estimations derived by TelomereHunter were in good agreement with experimental qPCR-based measurements, confirming earlier reports on the consistency of these methods [18]. This is highly relevant for clinical bioinformatics settings in which only NGS data is available for precision oncology diagnosis. Notably, the in silico estimations correlated better with each of the experimental methods than qPCR and TRF measurements with each other. Moreover, it is important to differentiate telomere content from telomere length. Telomere content is an observable value that describes the abundance of telomeric reads in a sample. To derive an estimate of the average telomere length from a telomere content estimate additional information such as the ploidy of the sample genome and the presence of extrachromosomal telomeric DNA have to be taken into account. TelomereHunter is not intended to provide telomere length estimates.

The exemplary results of an ALT-positive and an ALT-negative brain cancer case illustrate how a number of recent discoveries [21] are reflected in the output of TelomereHunter. The distribution of TVRs within individual reads and the quantification of singleton TVRs significantly improves the identification and study of ALT-positive cancer genomes as compared to a simple comparison of telomere content.

We have also profiled how telomere content quantification is influenced by different sequencing protocols. The absolute number of extracted intratelomeric reads in a sample is proportional to the robustness of the telomere content estimations. Here, the average of this value was 32-fold and 111-fold lower respectively for tumor and control samples in the WES cohort than for the WGS samples (Additional file 1: Table S4). Under a standard WES protocol, the main source of telomeric reads are actually unselected off-target reads. Interestingly, this implies a negative correlation between the quality of the target capture and the accuracy of the telomere content quantification. The development of telomere-sensitive WES protocols through the explicit addition of telomeric bait sequences would be a logical extension to compensate this shortcoming. We additionally observe an improved correlation of WES-based estimations and telomere qPCR quantification through normalization with a control sample, which may be related to cancellation of batch effects assuming that tumor and control samples were sequenced simultaneously. This observation advocates for the usage of matched controls especially for WES datasets. The comparison of software runs with and without alignment-based read filtering shows that telomere content estimation is slightly improved by removing reads that uniquely align to genomic coordinates. As the TRPM count from this source remains relatively constant, it contributes stronger to the overall count of telomeric reads in samples with short telomeres. Hence, these samples profit more strongly from the alignment-based filter. It is of even higher relevance for the analysis of TVRs in ALT-positive samples, as telomeric reads of subtelomeric and intra-chromosomal origin have a much higher heterogeneity in TVR content (Additional file 1: Figure S1) and thus partially mask the TVR signature of ALT-positive samples.

WGBS data showed acceptable correlations to WGS data concerning telomere content quantification but suffered from the absence of matched controls in our study. Furthermore, information on two relevant TVRs, namely TCAGGG and TTCGGG [20, 21], was lost in WGBS data due to the bisulfite conversion. Therefore, crucial information on telomere composition may be underrepresented in a WGBS-only study design.

## Conclusions

In this study, we have introduced TelomereHunter as a software tool for analysis of telomere content and composition, and have tested it on data from different next-generation sequencing protocols. Computational telomere content quantification from WGS data in cancer genome analysis was robust and showed excellent correlations with the experimental analysis. Moreover, we found that WGBS and WES data were also suited for telomere content quantification. WES is sensitive to batch effects and benefits from the availability of matched controls for telomere content normalization. In summary, TelomereHunter provides an in-depth characterization of telomere features and their deregulation in cancer cells from different types of sequencing readouts. It largely facilitates retrieving this information in the cohort-based analyses of cancer genomes. Thus, TelomereHunter extends the computational methods available for data mining of the increasing amount of next-generation sequencing data entering clinical routine towards novel schemes for patient stratification based on telomere features.

## Methods

### TelomereHunter implementation

TelomereHunter is written as a python package and takes BAM files of single samples or matched tumor and control pairs as input. Several parameters can be set by the user with the default settings and workflow being described in the following. In the first step of TelomereHunter, telomere reads containing at least *n* non-consecutive repeats (t-, c-, g- or j-type) are extracted (Fig. 1a). *n* is calculated for each read depending on the read length with the following formula: n = floor(read length · 0.06). The criterion of searching for six non-consecutive repeats in 100 bp reads has been proposed previously [2] and was also found suitable for the data presented in the present study.

In the second step, the extracted reads are categorized depending on the alignment coordinates and mapping quality. If reads are properly paired, the mapping position of the mate is considered for the sorting. In short, reads mapping to intrachromosomal regions, i.e. all chromosome bands except the first or last band, are defined as intrachromosomal reads. The subtelomeric fraction comprises telomeric reads mapped to the first or last band of a chromosome. Telomeric reads from paired-end data are classified as junction spanning if one mate maps to a first or last chromosome band and the other mate is unmapped. All unmapped reads or reads with a mapping quality lower than the defined threshold (default: 8) are categorized as intratelomeric.

The telomere content is calculated as the fraction of intratelomeric reads per million reads. To account for GC biases in sequencing data, TelomereHunter determines a GC-corrected telomere content: Instead of normalizing by the total number of reads in the sample, the intratelomeric reads are divided by the number of reads with a GC content between 48 and 52%, which is similar to that of the canonical t-type repeat and has been suggested for the normalization of telomeric reads [14].

TVRs are quantified by searching for NNNGGG hexamers in the intratelomeric reads. To avoid counting of sequencing errors, only hexamers with base qualities of at least 20 at every position are considered. The TVR counts are normalized to the total number of intratelomeric reads in the sample.

Next, TelomereHunter extracts the 18 bp sequences on either side of predefined TVR types by the user and counts all occurring combinations. Using default settings, this TVR context analysis is done for the ten most common TVRs found in a pan-cancer telomere study [21]. A particular focus is placed on singletons [(TTAGGG)_3_-NNNGGG-(TTAGGG)_3_], whose counts are normalized by the total number of reads in the sample. The output of TelomereHunter includes several diagrams visualizing the results (see Figs. 2 and 3 and Additional file 1: Figure S1 for examples).

### Whole genome sequencing

The WGS datasets analyzed in this study were obtained from the ICGC PedBrain Tumor project. Matching tumor and control samples were collected according to ICGC guidelines. The DNA libraries were prepared using Illumina paired-end sample preparation protocols and sequencing was performed on Genome Analyzer IIx and Illumina HiSeq 2000 instruments as previously described [22, 23]. Reads were aligned to the GRCh37 reference from 1000 Genomes project using bwa-mem version 0.7.8 with the option -T 0.

### Whole exome sequencing

The leiomyosarcoma WES datasets were obtained from a study by Chudasama *et al.* [24]. Matching tumor and control samples were collected according to World Health Organization criteria. Exomes were captured using SureSelect Human All Exon V5 + UTRs in-solution capture reagents (Agilent) and paired-end sequencing (2 × 101 bp) was performed with an Illumina HiSeq 2500 instrument as described previously [24]. Reads were aligned to the GRCh37 reference from 1000 Genomes project using two different alignment algorithms (bwa-mem version 0.7.8 with the option -T 0 and bwa-aln version 0.6.2 with the maximum insert size set to 1000 bp). Duplicates were removed in the datasets aligned with bwa-aln using Picard tools (version 1.90). Five tumor/control sample pairs were additionally aligned to the hgGRCh38 reference genome using bwa-mem version 0.7.8 with the option -T 0.

### Whole genome bisulfite sequencing

The WGBS datasets were obtained from the ICGC PedBrain Tumor project. Tumor samples were collected according to ICGC guidelines. Sequencing and data processing were performed as described previously [27]. Briefly, the library preparation included bisulfite conversion after adaptor ligation and sequencing was carried out with an Illumina HiSeq 2000 machine. The data was processed using MethylCtools. The reads were aligned against a single index of both *in silico* bisulfite-converted strands of the human reference genome (hg19, NCBI build 37.1) using BWA version 0.6.1-r104 with the non-default parameters -q 20 -s.

### Computational telomere content estimation

Telomere content estimation with TelomereHunter was calculated with the default settings unless otherwise indicated. Telomere contents without GC correction were calculated by dividing the intratelomeric reads by all reads in the sample. Telomere contents without filtering of aligned reads were calculated by dividing all telomeric reads by the number of reads with a GC content of 48–52%. To use exclusively t-type repeats (TTAGGG) for read extraction, TelomereHunter was run with -r TTAGGG. To test the influence of different repeat thresholds on telomere content estimation, TelomereHunter was run with repeat thresholds from 2 to 16 using the -rt parameter.

In addition to the TelomereHunter analysis, telomere content was quantified with four other software tools. Reads with six TNAGGG repeats were extracted using Motif Counter (http://sourceforge.net/projects/motifcounter/) [10] with the parameters -s -u -q 0. TelSeq version 0.01 [14] was run using default settings and the mean telomere content of different read groups was used for the benchmark. Telomere contents were determined from FASTQ files using Computel version 0.4.1 [15] with default parameters, R version 3.3.1 and samtools version 1.6. Telomerecat version 3.2 [17] was used with default parameters. In addition to the physical telomere length determined by Telomerecat, a telomere content was calculated by normalizing the number of extracted fully telomeric reads (“F1 reads”) to the total number of reads in the sample.

### Telomere quantitative real-time PCR

Telomere qPCR was conducted essentially as described previously [28, 29]. In short, 10 ng DNA, 1X LightCycler 480 SYBR Green I Master, 500 nM forward primer and 500 nM reverse primer were added per 10 μl reaction. The primer sequences were: telo fwd, 5′-CGGTTTGTTTGGGTTTGGGTTTGGGTTTGGGTTTGGGTT-3′; and telo rev, 5′-GGCTTGCCTTACCCTTACCCTTACCCTTACCCTTACCCT-3′; 36B4 fwd, 5′-AGCAAGTGGGAAGGTGTAATCC-3′; and 36B4 rev, 5′-CCCATTCTATCATCAACGGGTACAA-3′. Cycling conditions (for both telomere and 36B4 products) were 10 min at 95 °C, followed by 40 cycles of 95 °C for 15 s and 60 °C for 1 min. A standard curve was used to determine relative quantities of telomere repeats (T) to those of the single copy gene (S, *36B4* gene, also known as *RPLP0*). The T/S ratio was calculated for each sample (tumor and control) separately. The log2 ratio of telomere content was determined by dividing the T/S ratio of the tumor sample by the T/S ratio of the control sample. The calculated log2 ratio represents the increase or decrease in telomere content in tumor versus control samples.

### C-circle assay

The C-circle assay was performed according the protocol of Henson *et al.* [13]. Briefly, 30 ng DNA was combined with 10 μl 2X Φ29 Buffer, 7.5 U Φ29 DNA polymerase (both NEB), 0.2 mg/ml BSA, 0.1% (v/v) Tween 20, 1 mM each dATP, dGTP and dTTP and incubated at 30 °C for 8 h followed by 20 min at 65 °C. Reactions without addition of polymerase (−pol) were included as controls. After addition of 40 μl 2X SSC, the amplified DNA was dot-blotted onto a 2X-SSC-soaked Roti-Nylon plus membrane (Carl Roth). The membrane was baked for 20 min at 120 °C and hybridized and developed using the TeloTAGGG Telomere Length Assay Kit (Roche). Chemiluminescent signals were detected using a ChemiDoc MP imaging system (Bio-Rad).

### Terminal restriction fragment analysis

For TRF analysis, 4.5 μg genomic DNA of tumor and blood (control) samples were used, except for the GBM38 tumor and MB175 control sample, of which only 2.2 μg and 1.6 μg DNA were available, respectively. Genomic DNA was digested with the restriction enzymes HinfI and RsaI overnight. The digested DNA was resolved on a 0.6% agarose gel (Biozym Gold Agarose) in 1X TAE buffer using the CHEF-DRII pulsed-field gel electrophoresis system (Bio-Rad) with the following settings: 4 V/cm, initial switch time 1 s, final switch time 6 s, and 13 h duration. Southern blotting and chemiluminescent detection was performed using the TeloTAGGG Telomere Length Assay Kit (Roche) according to the manufacturer’s instructions. The blot was visualized with a ChemiDoc MP imaging system (Bio-Rad). The telomere content in each lane was determined by calculating the sum of intensities in each lane normalized to the amount of DNA loaded. This correction may not be sufficient if the difference of loaded DNA is too large. For this reason, MB175 was excluded from the correlation analysis of TRF-derived telomere content. It is noted that qPCR and TRF differ with respect to the normalization between samples. For telomere qPCR, the telomere content is normalized to a single copy gene and thus has an internal control for the amount of DNA used. This control is lacking for the TRF analysis where only the total amount of DNA loaded is measured. Thus, the TRF analysis is more prone to errors that arise from differences in the amount of DNA between samples.

### Extraction of alignment categories

The number of reads in different alignment categories was extracted using samtools version 0.1.17. The number of supplementary alignments in a BAM file was extracted using samtools view -f 2048 -c. The number of unmapped reads was extracted using samtools view -F 2048 -f 4 -c. The mapping qualities of all non-supplementary alignments were extracted from the fifth field of the BAM file. The number of reads mapping to sequences other than the reference genome were extracted from all non-supplementary alignments for which the reference sequence name (third field of the BAM file) was not chromosome 1:22, X or Y.

## Additional file

Additional file 1: **Figure S1.** Distribution of intrachromosomal telomeric reads. **Figure S2**: C-circle assay and TRF analysis of nine pediatric brain tumor samples and matching controls. **Figure S3.** Validation and benchmark of software tools for telomere content quantification. **Figure S4.** Classification of telomeric reads using alignment information. **Figure S5.** Categorization of bwa-mem alignment scores. **Figure S6.** Impact of alignment algorithms, extraction and filtering of telomeric reads on telomere content estimations. **Figure S7.** Impact of reference genomes on telomere content estimations. **Figure S8.** Influence of the repeat threshold parameter on telomere content estimation. **Table S1.** Parameters for TelomereHunter. **Table S2.** Description of TelomereHunter output files. **Table S3.** Run times and maximum memory usage of TelomereHunter. **Table S4.** Mean amount of intratelomeric reads. (PDF 453 kb)

## Abbreviations

c-type: TCAGGG
g-type: TGAGGG
j-type: TTGGGG
log_2_ T/C: Telomere content tumor/control log2 ratios
TRF: Terminal restriction fragment analysis
TRPM: Telomeric reads per GC content-matched million reads
t-type: TTAGGG
TVR: Telomeric variant repeat
WES: Whole exome sequencing
WGBS: Whole genome bisulfite sequencing
WGS: Whole genome sequencing

## References

[1] Coleman J, Baird DM, Royle NJ (1999). The plasticity of human telomeres demonstrated by a hypervariable telomere repeat array that is located on some copies of 16p and 16q. Hum Mol Genet.

[2] Lee M, Hills M, Conomos D, Stutz MD, Dagg RA, Lau LM (2014). Telomere extension by telomerase and ALT generates variant repeats by mechanistically distinct processes. Nucleic Acids Res.

[3] Varley H, Pickett HA, Foxon JL, Reddel RR, Royle NJ (2002). Molecular characterization of inter-telomere and intra-telomere mutations in human ALT cells. Nat Genet.

[4] Harley CB, Futcher AB, Greider CW (1990). Telomeres shorten during ageing of human fibroblasts. Nature..

[5] d’Adda di Fagagna F, Reaper PM, Clay-Farrace L, Fiegler H, Carr P, Von Zglinicki T, Saretzki G, Carter NP, Jackson SP (2003). A DNA damage checkpoint response in telomere-initiated senescence. Nature..

[6] Shay JW, Wright WE (2005). Senescence and immortalization: role of telomeres and telomerase. Carcinogenesis..

[7] Kim NW, Piatyszek MA, Prowse KR, Harley CB, West MD, Ho PL (1994). Specific association of human telomerase activity with immortal cells and cancer. Science..

[8] Bryan TM, Englezou A, Dalla-Pozza L, Dunham MA, Reddel RR (1997). Evidence for an alternative mechanism for maintaining telomere length in human tumors and tumor-derived cell lines. Nat Med.

[9] Feng J, Funk WD, Wang SS, Weinrich SL, Avilion AA, Chiu CP, Adams RR, Chang E, Allsopp RC, Yu J (1995). The RNA component of human telomerase. Science..

[10] Conomos D, Stutz MD, Hills M, Neumann AA, Bryan TM, Reddel RR (2012). Variant repeats are interspersed throughout the telomeres and recruit nuclear receptors in ALT cells. J Cell Biol.

[11] Shay JW (2016). Role of telomeres and telomerase in aging and Cancer. Cancer Discov.

[12] Aubert G, Hills M, Lansdorp PM (2012). Telomere length measurement-caveats and a critical assessment of the available technologies and tools. Mutat Res.

[13] Henson JD, Cao Y, Huschtscha LI, Chang AC, Au AY, Pickett HA, Reddel RR (2009). DNA C-circles are specific and quantifiable markers of alternative-lengthening-of-telomeres activity. Nat Biotechnol.

[14] Ding Z, Mangino M, Aviv A (2014). UK10K consortium, Spector T, Durbin R. estimating telomere length from whole genome sequence data. Nucleic Acids Res.

[15] Nersisyan L, Arakelyan A (2015). Computel: computation of mean telomere length from whole-genome next-generation sequencing data. PLoS One.

[16] Parker M, Chen X, Bahrami A, Dalton J, Rusch M, Wu G (2012). Assessing telomeric DNA content in pediatric cancers using whole-genome sequencing data. Genome Biol.

[17] Farmery JHR, Smith ML, Lynch AG, NIHR BioResource – Rare Diseases (2018). Telomerecat: a ploidy-agnostic method for estimating telomere length from whole genome sequencing data. Sci Rep.

[18] Lee M, Napier C, Yang F, Arthur J, Reddel R, Pickett H (2017). Comparative analysis of whole genome sequencing-based telomere length measurement techniques. Methods..

[19] Barthel Floris P, Wei Wei, Tang Ming, Martinez-Ledesma Emmanuel, Hu Xin, Amin Samirkumar B, Akdemir Kadir C, Seth Sahil, Song Xingzhi, Wang Qianghu, Lichtenberg Tara, Hu Jian, Zhang Jianhua, Zheng Siyuan, Verhaak Roel G W (2017). Systematic analysis of telomere length and somatic alterations in 31 cancer types. Nature Genetics.

[20] Lee M, Teber ET, Holmes O, Nones K, Patch A-M, Dagg RA (2018). Telomere sequence content can be used to determine ALT activity in tumours. Nucleic Acids Res.

[21] Sieverling L, Hong C, Koser SD, Ginsbach P, Kleinheinz K, Hutter B, et al. Genomic footprints of activated telomere maintenance mechanisms in cancer. Nat Commun. 2018; In press. 10.1038/s41467-019-13824-9 PMC7002710 32024817

[22] Bender S, Gronych J, Warnatz H-J, Hutter B, Gröbner S, Ryzhova M (2016). Recurrent MET fusion genes represent a drug traget in pediatric glioblastoma. Nat Med.

[23] Jones DT, Jager N, Kool M, Zichner T, Hutter B, Sultan M (2012). Dissecting the genomic complexity underlying medulloblastoma. Nature.

[24] Chudasama P, Mughal SS, Sanders MA, Hübschmann D, Chung I, Deeg KI, et al. Integrative genomic and transcriptomic analysis of leiomyosarcoma. Nat Commun. 2018;9:144. 10.1038/s41467-017-02602-0 PMC5762758 29321523

[25] Li H, Durbin R (2009). Fast and accurate short read alignment with burrows-wheeler transform. Bioinformatics..

[26] Li H, Durbin R. Fast and accurate long-read alignment with burrows-wheeler transform. Bioinformatics. 2010; Epub. 10.1093/bioinformatics/btp698 PMC2828108 20080505

[27] Hovestadt V, Jones DTW, Picelli S, Mang W, Kool M, Northcott PA (2014). Decoding the regulatory landscape of medulloblastoma using DNA methylation sequencing. Nature..

[28] Cawthon RM (2002). Telomere measurement by quantitative PCR. Nucleic Acids Res.

[29] O’Callaghan N, Dhillon V, Thomas P, Fenech M (2008). A quantitative real-time PCR method for absolute telomere length. Biotechniques.

