# Supplementary material for: TelomereHunter – in silico estimation of telomere content and composition from cancer genomes
Source: BMC Bioinformatics. 2019 May 28;20:272. doi: 10.1186/s12859-019-2851-0 (PMC6540518; doi:10.1186/s12859-019-2851-0)
Supplement: Supplementary file 1 — Figure S1. Distribution of intrachromosomal telomeric reads. Figure S2: C-circle assay and TRF analysis of nine pediatric brain tumor samples and matching controls. Figure S3. Validation and benchmark of software tools for telomere content quantification. Figure S4. Classification of telomeric reads using alignment information. Figure S5. Categorization of bwa-mem alignment scores. Figure S6. Impact of alignment algorithms, extraction and filtering of telomeric reads on telomere content estimations. Figure S7. Impact of reference genomes on telomere content estimations. Figure S8. Influence of the repeat threshold parameter on telomere content estimation. Table S1. Parameters for TelomereHunter. Table S2. Description of TelomereHunter output files. Table S3. Run times and maximum memory usage of TelomereHunter. Table S4. Mean amount of intratelomeric reads. (PDF 453 kb) [file 12859_2019_2851_MOESM1_ESM.pdf]

# TelomereHunter – *in silico* estimation of telomere content and composition from cancer genomes

**Supplementary Data**

Feuerbach *et al.*

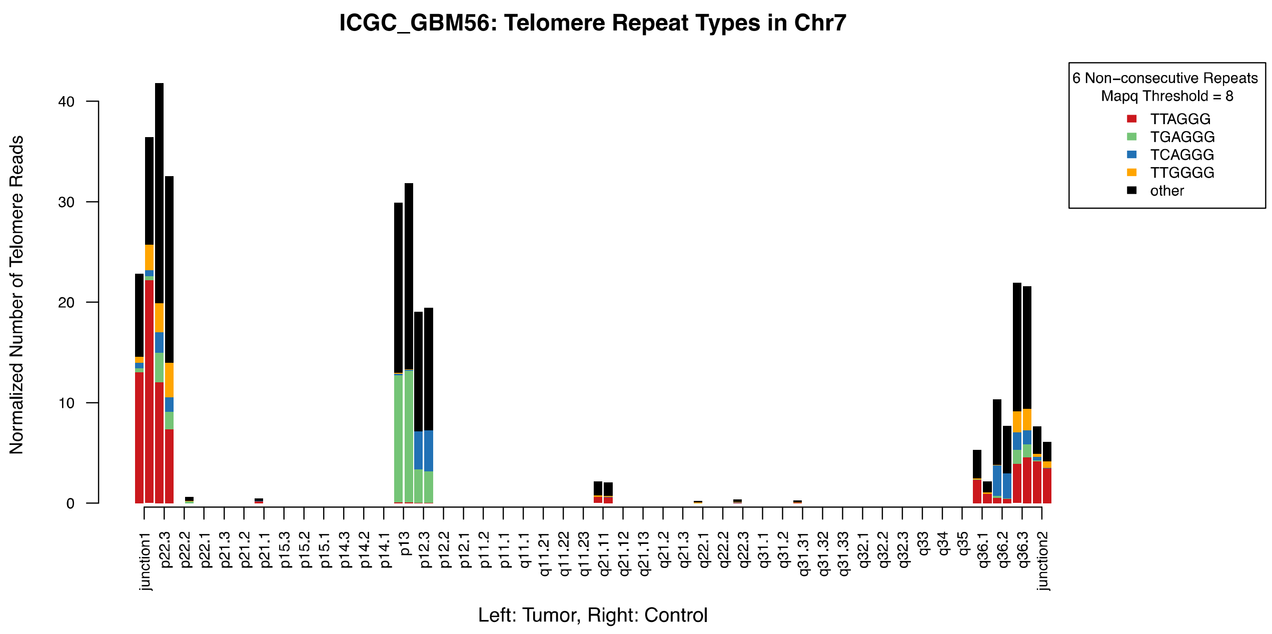


**Figure S1: Distribution of intrachromosomal telomeric reads.**
For each chromosome, the normalized number of telomere reads falling into each chromosome band is displayed. Junction spanning reads are shown as junction1 for the p-arm and junction2 for the q-arm. The color code indicates the abundance of particular hexameric-repeat units.

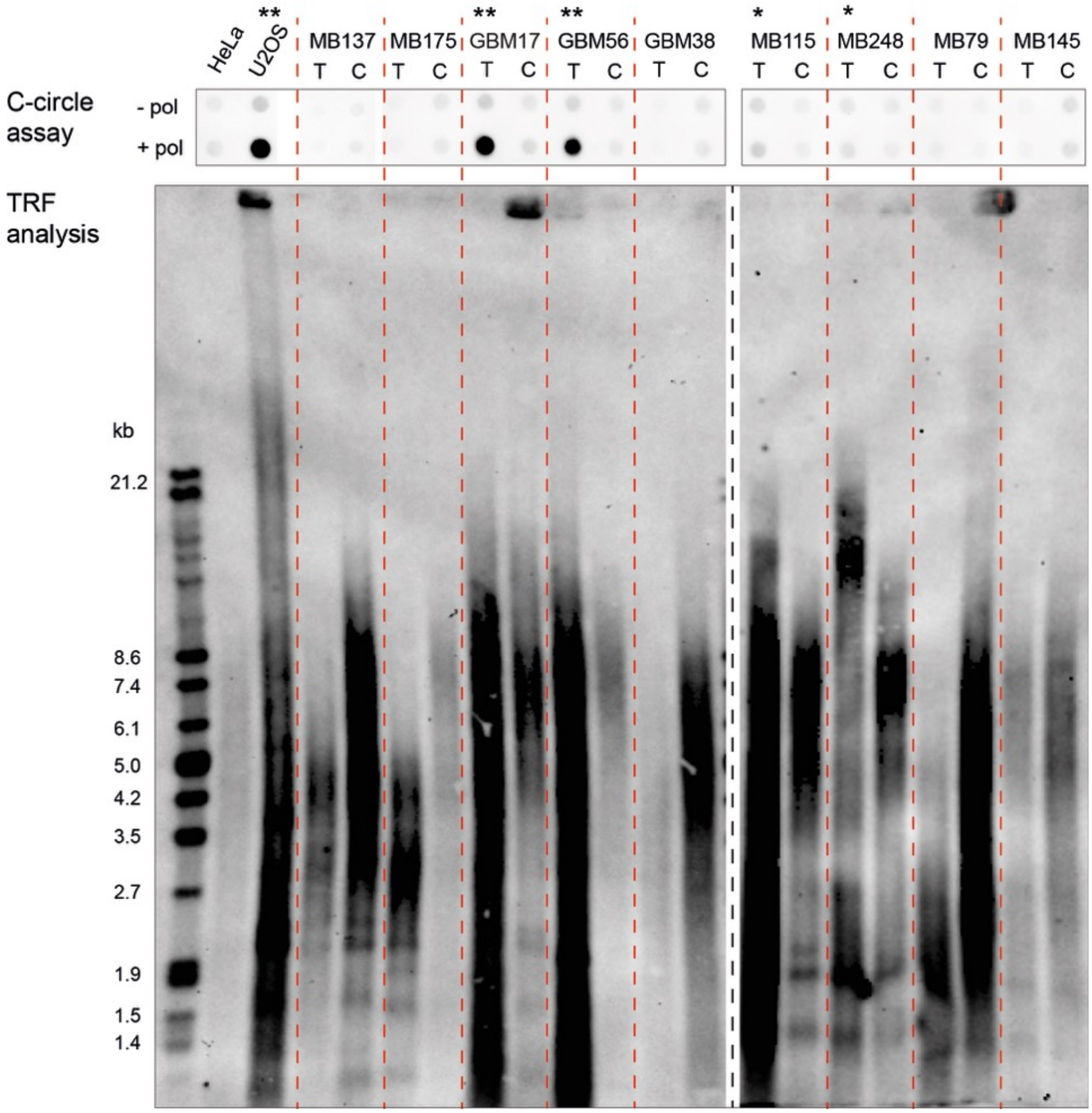


**Figure S2: C-circle assay and TRF analysis of nine pediatric brain tumor samples (T) and matching controls (C).**

The ALT-negative HeLa and the ALT-positive U2OS cell line were included as references. ALT-positive samples are highlighted by asterisks. * ALT-positive in TRF blot, ** ALT-positive in TRF blot and C-circle assay

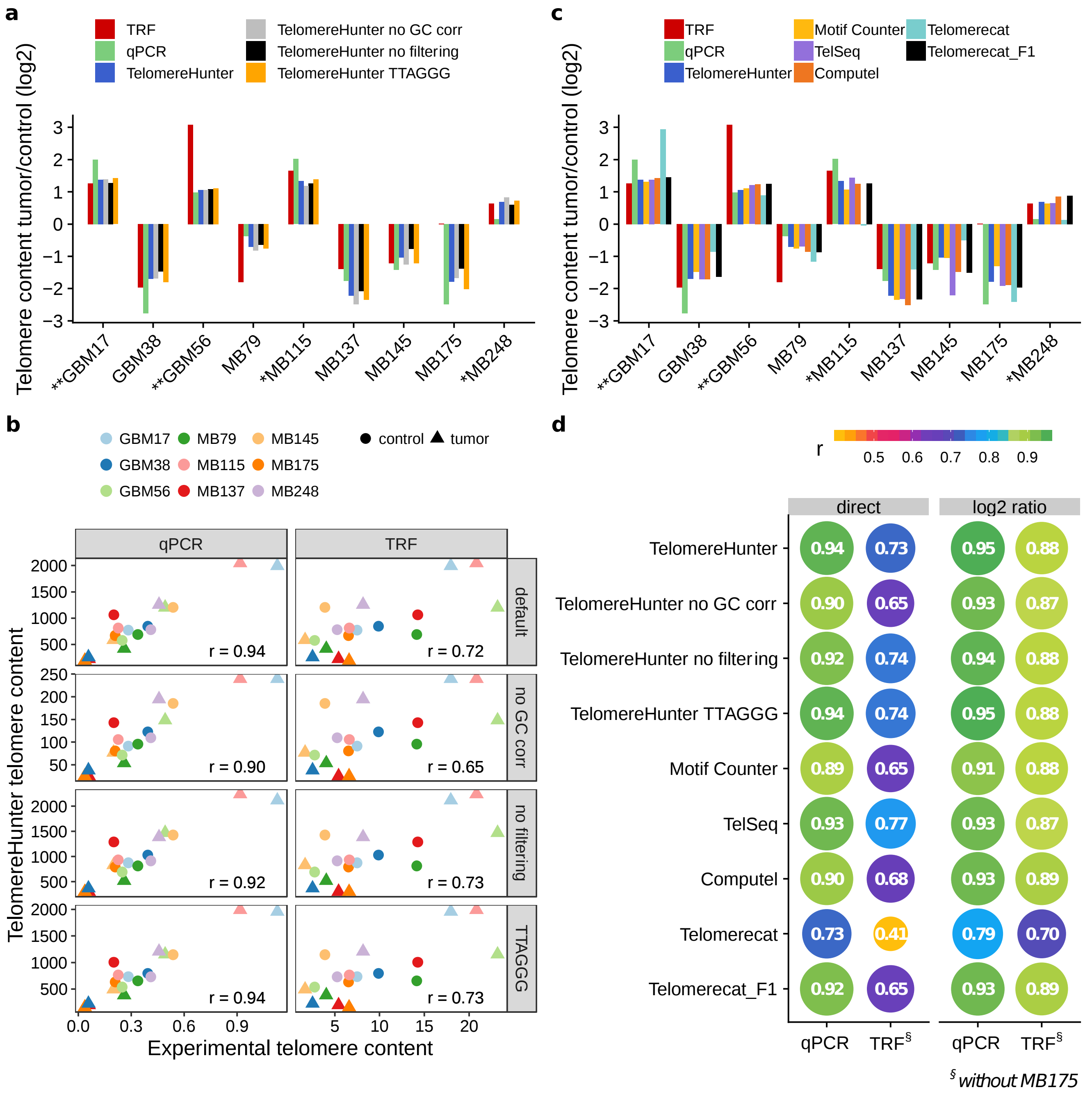


**Figure S3: Validation and benchmark of software tools for telomere content quantification.** Telomere content estimation with TelomereHunter is run in four different modes: (i) default configuration, (ii) without GC correction, (iii) no filtering of aligned reads and (iv) using exclusively t-type repeats (TTAGGG) for read extraction. (a) The telomere content $log_2$ *T/C* estimated by TRF, qPCR and TelomereHunter. (b) Pearson correlation coefficients of telomere content estimated by TelomereHunter and the telomere content measured by TRF and qPCR for individual tumor and control samples. Experimental telomere content values represent the summed intensities per ▯g of sDNA for TRF analysis and the telomere to single copy gene (T/S) ratios for qPCR. (c) The telomere content $log_2$ *T/C* determined by TRF, qPCR and different software tools are shown. (d) Pearson correlation coefficients of telomere contents determined by different software tools and qPCR and TRF for the

nine validation sample pairs. Correlation of individual samples is shown on the left, correlation of $log_2$ *T/C* is shown on the right. MB175 was excluded from the correlation with TRF, as different amounts of DNA were used in the experimental setup of this sample pair leading to difficulties for telomere content estimation. * ALT-positive in TRF blot, ** ALT-positive in TRF blot and C-circle assay

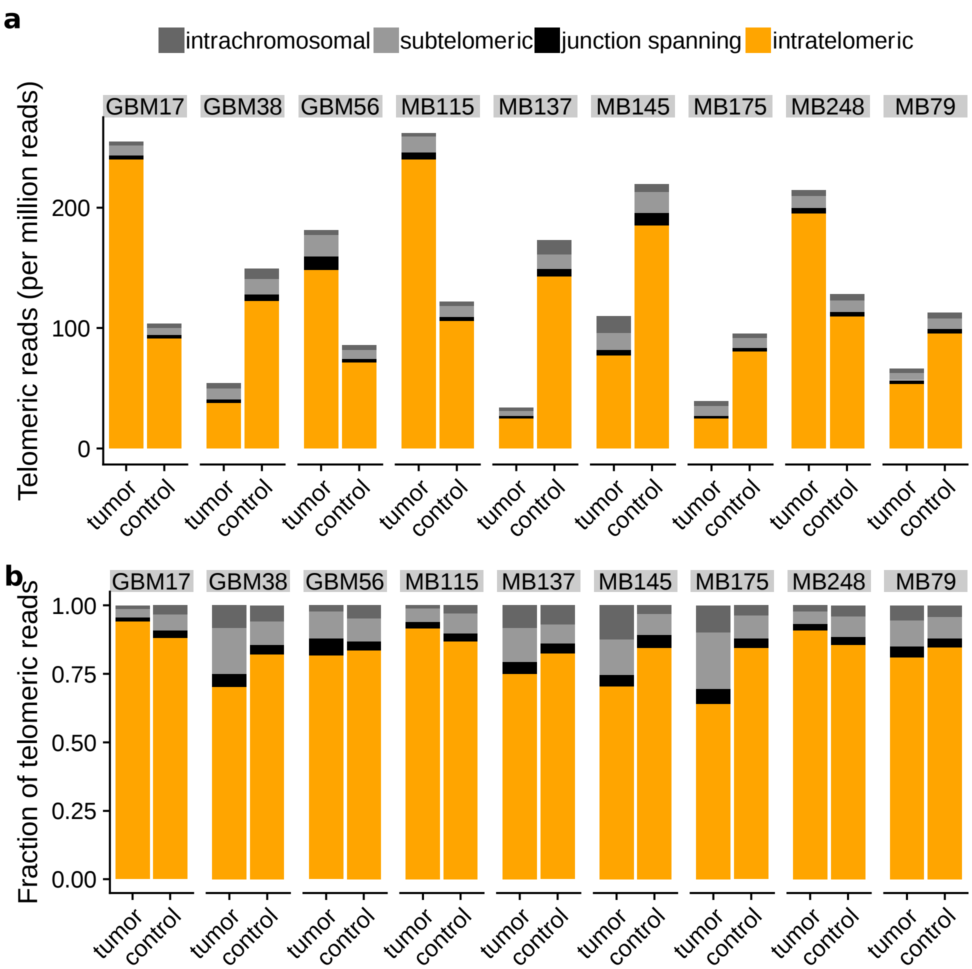


**Figure S4: Classification of telomeric reads using alignment information.**
The amount of telomeric reads falling into each of the four alignment-based categories distinguished by TelomereHunter is displayed for the nine patients of the validation set. (a) Absolute read counts per million total reads. (b) Relative proportion of each fraction.

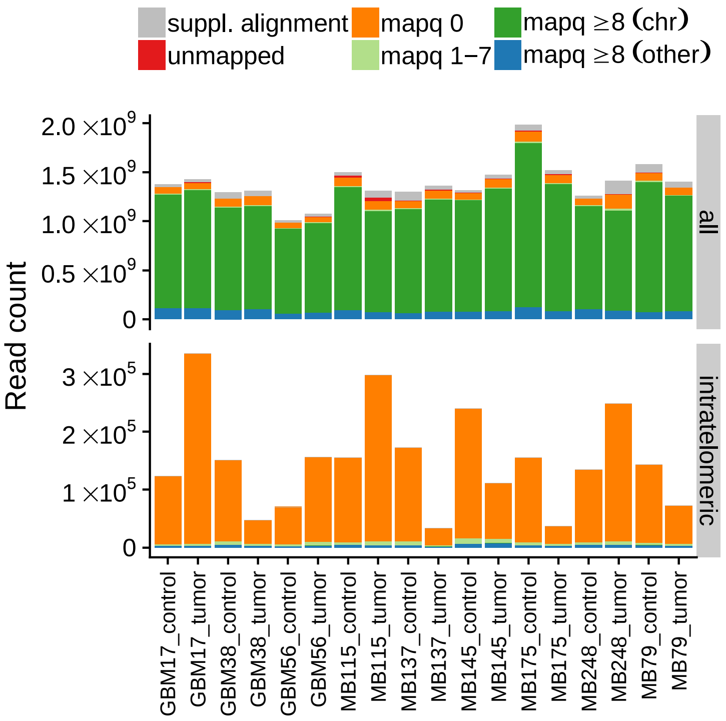


**Figure S5: Categorization of bwa-mem alignment scores.**
The distribution of alignment categories among the complete cancer genomes (all) and the extracted intratelomeric fraction is displayed for the nine patients of the validation cohort. The samples were aligned with bwa-mem. Alignment categories shown here are supplementary alignments, unmapped reads, and alignments with mapping qualities of 0, 1-7 or at least 8. Alignments with a mapping quality of at least 8 were divided into those mapping to reference chromosomes (“chr”) and those that aligned to other sequences (“other”).

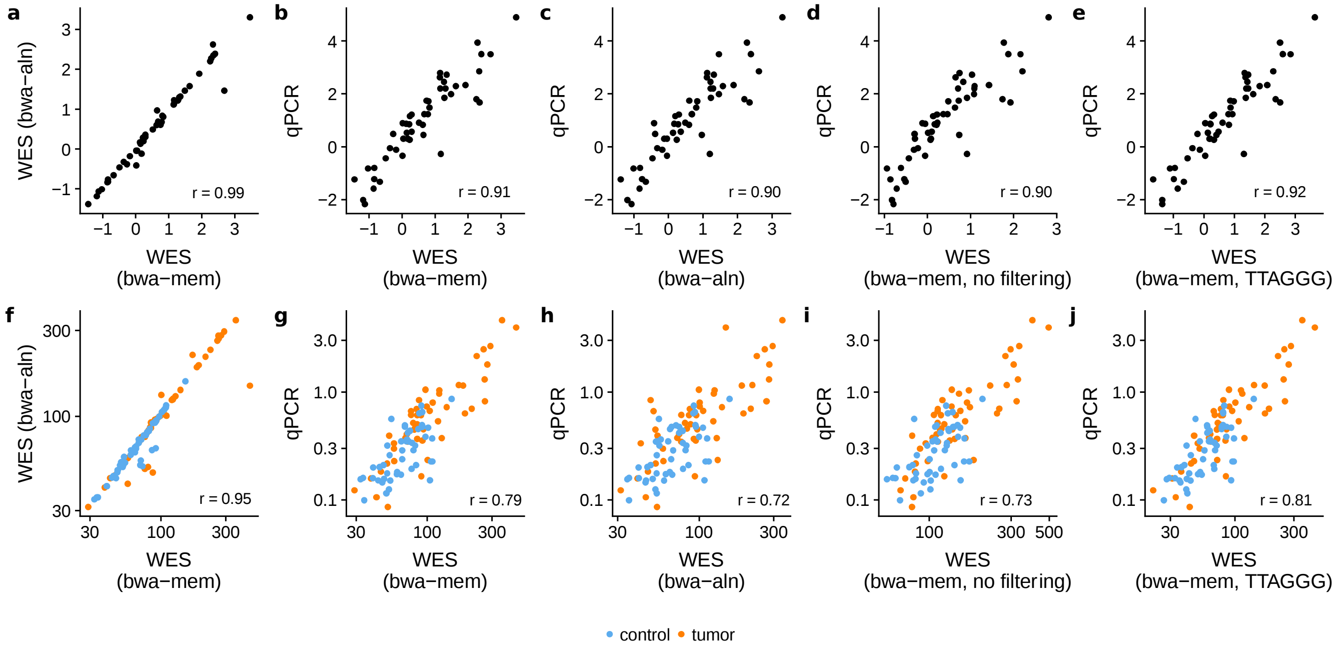


**Figure S6: Impact of alignment algorithms, extraction and filtering of telomeric reads on telomere content estimations.**

The impact of two alignment algorithms (bwa-mem and bwa-aln) on the TelomereHunter extraction and filtering steps was analyzed in context of the telomere content estimation using a dataset comprising WES tumor/control pairs from 49 leiomyosarcoma patients. The alignment algorithms (indicated in axis labels) were compared to telomere qPCR results. Telomere content from bwa-mem aligned reads was additionally calculated from all telomeric reads (no filtering) or by searching only for TTAGGG repeats in the telomere read extraction step of TelomereHunter. Spearman correlation coefficients are indicated. Panels (a)-(e) show the $log_2$ *T/C*, while panels (f)-(j) display separate estimates for tumor and control samples on a logarithmic scale.

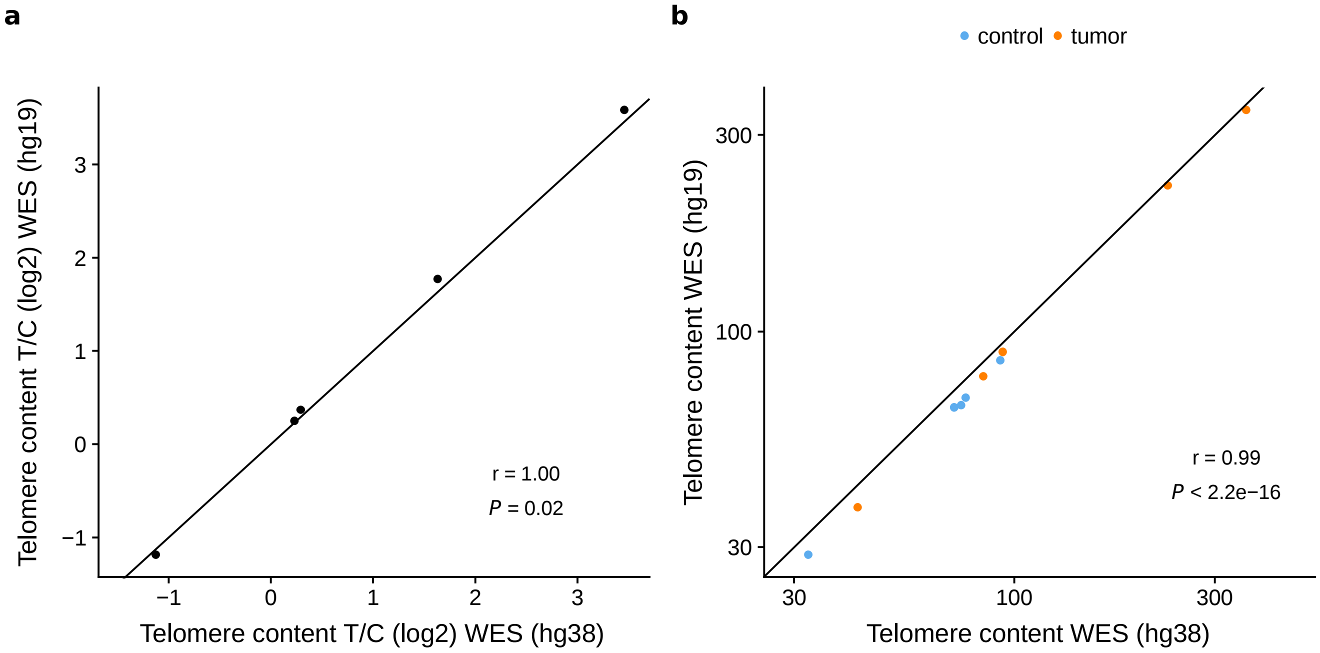


**Figure S7: Impact of reference genomes on telomere content estimations.**
The impact of bwa-mem alignment against two different versions of the human reference genome, hg19 and hg38, on telomere content estimation was estimated using five exemplary WES tumor/control pairs from leiomyosarcoma patients. (a) Correlation of telomere content *log2 T/C*. (b) Correlation of TelomereHunter results for individual tumor and control samples of the same patients (shown on a logarithmic scale). The Spearman correlation coefficients are indicated.

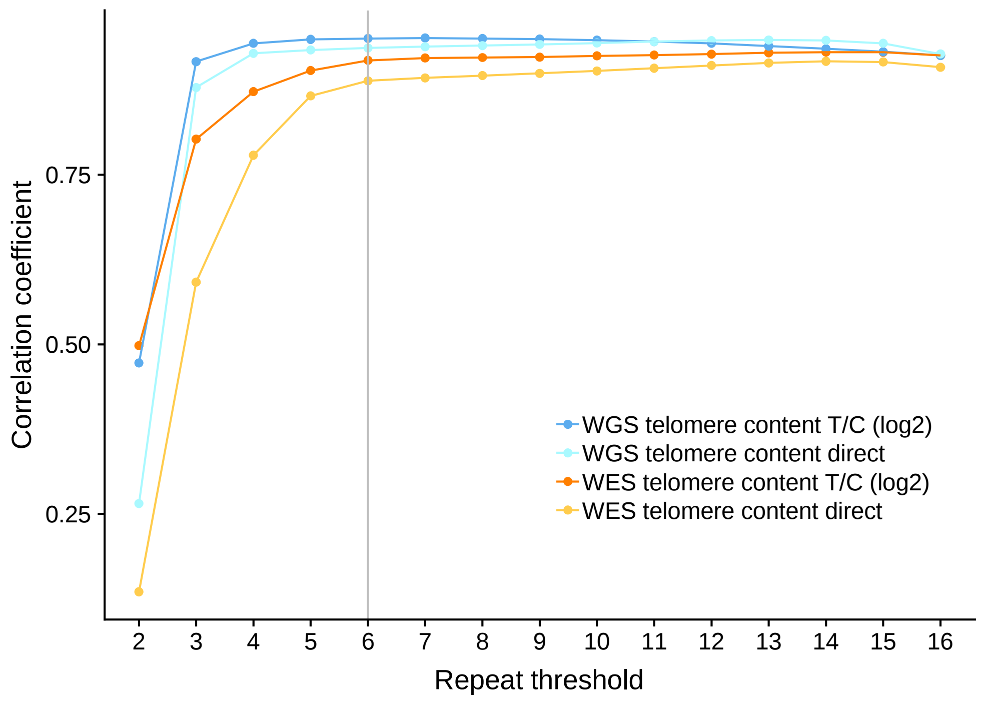


**Figure S8: Influence of the repeat threshold parameter on telomere content estimation.** Variations on the minimal telomeric repeat threshold (x-axis) impact the Pearson correlation of *in silico* telomere content estimations using TelomereHunter with telomere content estimated by qPCR measures (y-axis). The influence of different repeat thresholds was tested for 9 WGS tumor/control glioblastoma and medulloblastoma sample pairs and 49 WES tumor/control leiomyosarcoma sample pairs. For each cohort, the correlation was determined for the $log_2$ *T/C* and the individual samples (direct).

**Table S1: Parameters for TelomereHunter.**

| **Parameter** | **Description** |
|---|---|
| -h, --help | show help message |
| -ibt TUMOR_BAM, --inputBamTumor TUMOR_BAM | Path to the indexed input BAM file of the tumor sample. |
| -ibc CONTROL_BAM, --inputBamControl CONTROL_BAM | Path to the indexed input BAM file of the control sample. |
| -o OUTPUT_DIR, --outPath OUTPUT_DIR | Path to the output directory into which all results are written. |
| -p PID, --pid PID | Sample name used in output files and diagrams (required). |
| -b BANDING_FILE, --bandingFile BANDING_FILE | Path to a tab-separated file with information on chromosome banding. The first four columns of the table have to contain the chromosome name, the start and end position and the band name. The table should not have a header. If no banding file is specified, the banding information of hg19 will be used. |
| -rt REPEAT_THRESHOLD_SET, --repeatThreshold REPEAT_THRESHOLD_SET | The number of repeats needed for a read to be classified as telomeric. If no repeat threshold is defined, TelomereHunter will calculate the repeat_threshold depending on the read length with the following formula: repeat_threshold = floor(read_length * 6/100) |
| -rl, --perReadLength | Repeat threshold is set per 100 bp read length. The used repeat threshold will be: floor(read_length * repeat_threshold/100) E.g. Setting -rt 8 -rl means that 8 telomere repeats are required per 100 bp read length. If the read length is 50 bp, the threshold is set to 4. |
| -mqt MAPQ_THRESHOLD, --mappingQualityThreshold MAPQ_THRESHOLD | The mapping quality needed for a read to be considered as mapped (default = 8). |
| -d, --removeDuplicates | Reads marked as duplicates in the input bam file(s) are removed in the filtering step. |
| -r REPEATS, --repeats REPEATS | List of telomere repeat types to search for. Reverse complements are automatically generated and do not need to be specified! By default, TelomereHunter searches for t-, g-, c- and j-type repeats (TTAGGG TGAGGG TCAGGG TTGGGG). |
| -con, --consecutive | Search for consecutive repeats. |
| -gc1 LOWERGC, --lowerGC LOWERGC | Lower limit used for GC correction of telomere content. The value must be an integer between 0 and 100 (default = 48). |
| -gc2 UPPERGC, --upperGC UPPERGC | Upper limit used for GC correction of telomere content. The value must be an integer between 0 and 100 (default = 52). |
| -nf, --noFiltering | If the filtering step of TelomereHunter has already been run previously, skip this step. |
| -rc TVRS_FOR_CONTEXT, --repeatsContext TVRS_FOR_CONTEXT | List of telomere variant repeats for which to analyze the sequence context. Reverse complements are automatically generated and do not need to be specified! Counts for these telomere variant repeats (arbitrary and singleton context) will be added to the summary table. Default repeats: TCAGGG TGAGGG TTGGGG TTCGGG TTTGGG ATAGGG CATGGG CTAGGG GTAGGG TAAGGG). |
| -bp BP_CONTEXT, --bpContext BP_CONTEXT | Number of base pairs on either side of the telomere variant repeat to investigate. Please use a number that is divisible by 6. |
| -pl, --parallel | The filtering, sorting and estimating steps of the tumor and control sample are run in parallel. This will speed up the computation time of TelomereHunter. |
| -pff {pdf,png,svg,all}, --plotFileFormat {pdf,png,svg,all} | File format of output diagrams. Choose from pdf (default), png, svg or all (pdf, png and svg). |

| | |
|---|---|
| -p1, --plotChr | Make diagrams with telomeric reads mapping to each chromosome. |
| -p2, --plotFractions | Make a diagram with telomeric reads in each fraction (intrachromosomal, subtelomeric, junction spanning, intratelomeric). |
| -p3, --plotTelContent | Make a diagram with the gc corrected telomere content in the analyzed samples. |
| -p4, --plotGC | Make a diagram with GC content distributions in all reads and in intratelomeric reads. |
| -p5, --plotRepeatFreq | Make histograms of the repeat frequencies per intratelomeric read. |
| -p6, --plotTVR | Make plots for telomere variant repeats. |
| -p7, --plotSingleton | Make plots for singleton telomere variant repeats. |
| -p8, --plotNone | Do not make any diagrams. |
| -prc, --plotRevCompl | Distinguish between forward and reverse complement telomere repeats in diagrams. |

## Table S2: Description of TelomereHunter output files.

| | |
|---|---|
| **individual output files for tumor and control sample** | |
| | |
| **bam files** | |
| [sample_ID]_filtered.bam | all extracted telomere reads (sorted by position) |
| [sample_ID]_filtered_name_sorted.bam | all extracted telomere reads (sorted by read name) |
| [sample_ID]_filtered_intrachromosomal.bam | intrachromosomal telomere reads |
| [sample_ID]_filtered_subtelomeric.bam | subtelomeric telomere reads |
| [sample_ID]_filtered_junctionspanning.bam | junction spanning telomere reads |
| [sample_ID]_filtered_intratelomeric.bam | intratelomeric telomere reads |
| | |
| **tables** | |
| [sample_ID]_spectrum.tsv | number of telomere reads per chromosome band and their composition |
| [sample_ID]_readcount.tsv | total number of reads per chromosome band |
| [sample_ID]_repeat_frequency_per_intratelomeric_read.tsv | count table of telomere repeats per intratelomeric read |
| [sample_ID]_[tumor/control]_gc_content.tsv | GC content count table for all reads |
| [sample_ID]_intratelomeric_[tumor/control]_gc_content.tsv | GC content count table for intratelomeric reads |
| [sample_ID]_[tumor/control]_summary.tsv | summary of telomere content estimations |
| | |
| **TVRs** | |
| [sample_ID]_[tumor/control]_TVRs.txt | count, frequency and average base qualities of TVRs |
| | |
| **TVR_context** | |
| [sample_ID]_[tumor/control]_[TVR]_18bp_18bp_neighborhood.tsv | count and frequency of all TVR contexts (18 bp on either side of the TVR) |
| [sample_ID]_[tumor/control]_[TVR]_18bp_neighborhood_before.tsv | count and frequency of all TVR contexts (18 bp upstream of the TVR) |
| [sample_ID]_[tumor/control]_[TVR]_18bp_neighborhood_after.tsv | count and frequency of all TVR contexts (18 bp downstream of the TVR) |
| | |
| | |
| | |
| **joined output files for matched tumor and control samples** | note: these will also be produced for single samples if a control is not available |
| | |
| **tables** | |
| [sample_ID]_normalized_TVR_counts.tsv | TVR counts normalized in different ways |
| [sample_ID]_TVR_top_contexts.tsv | the most common sequence context for all TVRs |
| [sample_ID]_singletons.tsv | absolute and normalized singleton counts and the distance to the expected singleton log2 ratio |
| [sample_ID]_summary.tsv | summary of the most important produced values |
| | |
| **plots** | |
| [sample_ID]_telomere_content.pdf | telomere content and composition |
| [sample_ID]_sorted_telomere_read_counts.pdf | read counts and composition of different telomere read groups |
| [sample_ID]_[chromosome].pdf | count and composition of telomere reads mapping to chromosome bands |

| | |
|---|---|
| [sample_ID]_gc_content.pdf | GC content distribution in intratelomeric and in all reads |
| [sample_ID]_hist_telomere_repeats_per_intratelomeric_read.pdf | frequency of telomere repeats in intratelomeric reads |
| [sample_ID]_TVR_barplot.pdf | normalized TVR counts and log2 T/C |
| [sample_ID]_TVR_scatterplot.pdf | scatterplot of TVR counts in the tumor and control sample |
| [sample_ID]_singletons.pdf | raw and normalized singleton counts, log2 T/C and distance to expected singleton counts |
| [sample_ID]_all_plots_merged.pdf | all plots merged into a single PDF document |
| [sample_ID]_telomerehunter_summary_plot.pdf | summary of the TelomereHunter analysis |

**Table S3: Run times and maximum memory usage of TelomereHunter.**
TelomereHunter was run for an exemplary WGS, WES and WGBS and sample.

| Data type | File size | Total number of reads | Run time | Max. memory |
|---|---|---|---|---|
| WES | 23G | 308,022,212 | 1:45 h | 89.3 mb |
| WGS | 80G | 1,050,171,464 | 6:53 h | 79.5 mb |
| WGBS | 176G | 2,355,508,254 | 13:55 h | 504.9 mb |

**Table S4: Mean amount of intratelomeric reads.**
The mean total number of intratelomeric and normalized number of intratelomeric reads of the here analyzed WES (bwa mem aligned), WGS and WGBS cohorts are shown.

| | | Mean intratel_reads | Mean intratel_reads/all_reads*1 Mio |
|---|---|---|---|
| **WES (LMS)** | **tumor (n = 49)** | 2985 | 21 |
| | **control (n = 49)** | 1133 | 12 |
| **WGS (MB)** | **tumor (n = 34)** | 94618 | 70 |
| | **control (n = 34)** | 125837 | 86 |
| **WGBS (MB)** | **tumor (n = 34)** | 272667 | 218 |
